# Trafficking of Sendai Virus Nucleocapsids Is Mediated by Intracellular Vesicles

**DOI:** 10.1371/journal.pone.0010994

**Published:** 2010-06-07

**Authors:** Raychel Chambers, Toru Takimoto

**Affiliations:** Department of Microbiology and Immunology, University of Rochester Medical Center, Rochester, New York, United States of America; University of Georgia, United States of America

## Abstract

**Background:**

Paramyxoviruses are assembled at the plasma membrane budding sites after synthesis of all the structural components in the cytoplasm. Although viral ribonuclocapsid (vRNP) is an essential component of infectious virions, the process of vRNP translocation to assembly sites is poorly understood.

**Methodology/Principal Findings:**

To analyze real-time trafficking of vRNPs in live infected cells, we created a recombinant Sendai virus (SeV), rSeVLeGFP, which expresses L protein fused to enhanced green fluorescent protein (eGFP). The rSeVLeGFP showed similar growth kinetics compared to wt SeV, and newly synthesized LeGFP could be detected as early as 8 h postinfection. The majority of LeGFP co-localized with other components of vRNPs, NP and P proteins, suggesting the fluorescent signals of LeGFP represent the locations of vRNPs. Analysis of LeGFP movement using time-lapse digital video microscopy revealed directional and saltatory movement of LeGFP along microtubules. Treatment of the cells with nocodazole restricted vRNP movement and reduced progeny virion production without affecting viral protein synthesis, suggesting the role of microtubules in vRNP trafficking and virus assembly. Further study with an electron microscope showed close association of vRNPs with intracellular vesicles present in infected cells. In addition, the vRNPs co-localized with Rab11a protein, which is known to regulate the recycling endocytosis pathway and Golgi-to-plasma membrane trafficking. Simultaneous movement between LeGFP and Rab11a was also observed in infected cells, which constitutively express mRFP-tagged Rab11a. Involvement of recycling endosomes in vRNP translocation was also suggested by the fact that vRNPs move concomitantly with recycling transferrin labeled with Alexa 594.

**Conclusions/Significance:**

Collectively, our results strongly suggest a previously unrecognized involvement of the intracellular vesicular trafficking pathway in vRNP translocation and provide new insights into the transport of viral structural components to the assembly sites of enveloped viruses.

## Introduction

Sendai virus (SeV) is a pneumotropic murine pathogen in the *Paramyxoviridae* family, which includes many clinically important human pathogens [1]. It is an enveloped negative-strand RNA virus and encodes 6 major structural proteins: two glycoproteins (HN and F), the matrix (M) protein, the nucleoprotein (NP), and the phosphoprotein (P) and large (L) proteins, which make up the polymerase complex. The viral RNA (vRNA) is encapsidated by NP, and the P-L polymerase complex associates with the vRNA-NP complex to form ribonucleocapsids (vRNPs). The polymerase complex is responsible for transcription and replication of the viral genome, which occurs completely in the cytoplasm of infected cells. Newly synthesized structural components including vRNPs are transported to the plasma membrane, where progeny viruses are assembled and formed by budding. At the budding sites, vRNPs are wrapped by a lipid bilayer envelope containing an internal M layer and the HN and F spike proteins, which are exposed on the virion surface [1].

Although components of virions must translocate to budding sites for progeny virion formation, little is known about the mechanism of how viral structural components are transported to the plasma membrane for assembly. Among the viral structural proteins, M protein, which lines the inner surface of the host cell's plasma membrane, is well established as playing a key role in virion assembly. Expression of M protein alone induces budding, and it interacts with specific envelope glycoproteins and vRNPs [2]. The association between SeV M and glycoproteins seems to be required for M trafficking to budding sites, since restriction of glycoproteins at the Golgi compartment by monensin or low temperature incubation resulted in accumulation of M protein on Golgi membranes [3]. This suggests that M utilizes trafficking of envelope glycoproteins through the secretory pathway for its translocation to the plasma membrane. Similarly, a specific M-vRNP interaction is required for the uptake and incorporation of vRNPs into progeny virions at the plasma membrane [4], however, it is not clear whether vRNPs interact with M during translocation through the cytosol or upon reaching the plasma membrane.

Two cytoskeletal elements, the microtubule (MT) and actin networks, support motor protein-driven intracellular transport, including vesicles and organelles. In animal cells, MTs provide high-speed, long-range transport. Most intracellular transport occurs via the MT network. Some recent studies suggest that cellular MT motors catalyze the intracellular transport of viral core structures during the entry process of both DNA and RNA viruses; examples are herpes simplex virus [5], human cytomegalovirus [6], parvoviruses [7]–[8], human immunodeficiency virus [9], influenza A virus [10], and vesicular stomatitis virus (VSV) [11]. Using a yeast two-hybrid cDNA library screen, rabies virus P protein was found to interact strongly with the cytoplasmic dynein light chain, a component of both MT- and actin-based motors [12]–[14]. Moreover, the matrix protein present in the cytosolic Gag polyprotein of many retroviruses binds to kinesin-4 [15], and this interaction is suggested to convey subviral particles to the plasma membrane for virus budding. These studies suggest that the cellular trafficking system that utilizes MTs may have important roles for many viruses during assembly and egress. In the case of paramyxoviruses, however, little is known about the role of the MT network in virus assembly.

An additional facet of cellular trafficking involves transport of cellular cargo by vesicles. Vesicular transport of cargo within the cell is tightly regulated and characteristically is facilitated by the use of vesicles that bud from a donor compartment and fuse with an acceptor compartment. Vesicular transport specificity and fidelity are directed by vesicular membrane proteins and small molecular weight Rab GTPases [16]. Rab proteins are specific for various membranes, and the specificity is mediated by Rab interactions with effector molecules [17]–[18]. Two examples of Rab proteins, Rab8 and Rab11, have been shown to be vital for transporting cargo from the Golgi to the plasma membrane [19]–[21]. Inhibition of these proteins disrupts recycling endosome-dependent transport from the Golgi to the plasma membrane [21]. Rab8 has been shown to be involved in transport of the glycoproteins of VSV and Semliki Forest virus to the cell surface [22]–[23], while both Rab8 and Rab11 play a role in hantavirus release [24]. As with the role of MTs in transport of paramyxovirus RNPs to sites of assembly, the role of the vesicular trafficking pathways in paramyxovirus assembly has not been identified.

The real-time visualization of virus particles or viral structural components in live cells provides an abundance of information about the life cycle of the viruses. A widely used approach for the visualization of viral components is to rescue recombinant viruses which express a structural protein fused to a fluorescent protein, such as enhanced green fluorescent protein (eGFP). This approach has been successfully utilized to track virions or viral proteins in infected cells. To study the translocation process of vRNPs in live cells, we rescued a recombinant SeV, rSeVLeGFP, which expresses the L protein fused to eGFP. Analysis of cells infected with rSeVLeGFP showed vRNPs trafficking on the MT network. Disruption of MT structure by nocodazole immobilized vRNP movement and reduced the production of progeny virions without affecting viral protein levels in infected cells, suggesting that trafficking through MTs is required for the assembly of progeny virions. Additional study using electron microscopy (EM) revealed close association of vRNPs with intracellular vesicles, which appear to be abundant in virus-infected cells. The vRNPs in cells also co-localized with Rab11a, a known regulator of the recycling endosome pathway. In addition, concomitant movement of vRNPs with transferrin and with Rab11a was observed. Our data suggest that SeV vRNPs traffic through the cytoplasm using intracellular vesicles along the MT network, which is likely to lead to the translocation of vRNP to viral assembly/budding sites at the plasma membrane of infected cells.

## Results

### Rescue and characterization of rSeVLeGFP

**Figure 1:**
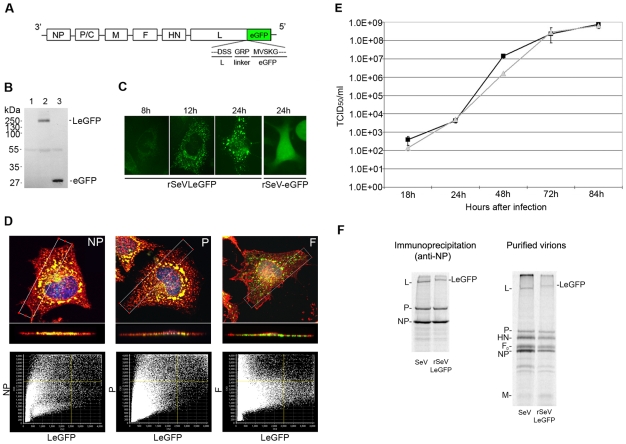
Rescue and characterization of rSeVLeGFP. (A) Schematic diagram of rSeVLeGFP genome. The rSeVLeGFP genome expresses eGFP (239 residues) fused to the C-terminus of the L protein (2,228 residues). (B) Western blot analysis of LeGFP expressed in infected cells. Lysates of mock- (lane 1), rSeVLeGFP- (lane 2), or rSeV-eGFP- (lane 3) infected cells were reacted with anti-GFP antibody. (C) Analysis of LeGFP localization in live cells. HeLa cells were infected with rSeVLeGFP and images of the cells were captured at the indicated h p.i. The same settings for the fluorescent microscope were used at each time point. (D) Co-localization of SeV NP, P and F proteins with LeGFP. HeLa cells were infected with rSeVLeGFP for 24 h. Fixed cells were stained for NP, P or F using specific Mabs. SeV NP, P or F are shown in red and LeGFP in green. Merged images in the top row are deconvoluted z-stack images of the xy plane, and the images in the second row are *z*-axis reconstructions, taken across the *xy* plane at the white boxed region of interest, shown in the top row. The bottom row of graphs represents overlapping degree of intensity scatterplots of each pixel in the image, as determined by the Olympus FV1000 software. Yellow lines represent the threshold set at 2500. (E) Growth kinetics of rSeVLeGFP as compared with wt SeV. LLC-MK_2_ cells were infected at MOI 0.01 with rSeVLeGFP (gray triangle) or wt SeV (closed square) and incubated at 34°C. Aliquots of infected-cell supernatants were collected and viral titers of supernatants were determined in LLC-MK_2_ cells. (F) Characterization of viral proteins in purified virions and cell lysates of infected cells. HeLa cells were infected with either SeV or rSeVLeGFP and labeled with ^35^S-Met/Cys. SeV or rSeVLeGFP vRNP complexes in infected cells were immunoprecipitated by anti-NP Mab and analyzed by SDS-PAGE. Virions in the supernatants were purified and analyzed by SDS-PAGE.

To visualize movement of vRNPs in the cytoplasm of infected cells, we fused SeV L protein gene with the eGFP gene (LeGFP), and cloned this fusion gene into the full genome SeV cDNA (Figure 1A). Using the reverse genetics rescue system, the recombinant virus rSeVLeGFP that expresses LeGFP was successfully rescued. The expression of the LeGFP protein was determined by Western blotting analysis, using an anti-GFP antibody. As a control for eGFP expression, we rescued another recombinant SeV, rSeV-eGFP, which expresses free eGFP that is not fused to any viral protein, from an additional gene inserted between the F and HN genes. As shown in Figure 1B, only a single specific band of LeGFP was detected, confirming that eGFP signals in infected cells represent LeGFP and not the non-fused eGFP, which could be produced by cellular proteases. We next determined the appearance of fluorescent signals at various times after infection. We used HeLa cells because they have an expansive cytoplasm and are known to support SeV growth [25]. HeLa cells infected with rSeVLeGFP at an MOI of 1.0 were observed with a fluorescent microscope at various times after infection (Figure 1C). Fluorescent signals of LeGFP could be detected as early as 8 h after infection, and by 12 h numerous individual dots of strong fluorescent signals were visible. At 24 h, in addition to the small dots, larger signals of fluorescence were also evident. The fluorescent punctae, which appeared in rSeVLeGFP-infected cells were clearly distinguishable from the diffuse signals of eGFP observed in cells infected with rSeV-eGFP that expresses non-tagged free eGFP from an additional gene (Figure 1C). L protein forms a polymerase complex with P protein and associates with nucleocapsids composed of viral RNA and NP. Using confocal microscopy, we next determined the co-localization of LeGFP with P and NP proteins to confirm that LeGFPs are associated with nucleocapsids. HeLa cells infected with rSeVLeGFP for 24 h were fixed, permeabilized and reacted with anti-NP, anti-P or anti-F monoclonal antibodies (Mab) followed by anti-mouse IgG-Texas Red. The results of the 3-D confocal analysis revealed that LeGFP co-localized well with P and NP, but not F proteins (Figure 1D). Detailed graphic analysis comparing the intensity of individual fluorescent pixels indicated that 91.5% and 77.9% of LeGFP co-localized with P and NP, respectively. These data contrasted distinctly to the limited co-localization calculated between LeGFP and F glycoprotein at around 25.3% (Figure 1D bottom panels), and confirm that the majority of LeGFPs detected in the cytoplasm are associated with viral nucleocapsids.

To ascertain whether the fusion of eGFP to the C-terminus of L protein would interfere with viral replication and growth, we compared the growth kinetics of wt SeV and rSeVLeGFP in LLC-MK_2_ cells. The cells were infected with the viruses at an MOI of 0.01 and cultured with trypsin for 84 h. The rSeVLeGFP grew to a similar titer as wt SeV in the cell line (Figure 1E), indicating the presence of eGFP at the C-terminus of L protein does not significantly inhibit the function of SeV L protein. Furthermore, we compared the production of the vRNP complex in wt SeV- and rSeVLeGFP-infected cells by radioimmunoprecipitation using anti-NP Mab. The vRNPs composed of NP, P and LeGFP were recovered from rSeVLeGFP-infected cells at a similar molecular ratio to that recovered from wt SeV-infected cells (Figure 1F left). Also, purified rSeVLeGFP virions contained vRNPs with a similar molecular ratio of NP, P and L as that detected from wt SeV (right panel). These results indicate that fusion of eGFP to the C-terminus of L protein does not significantly affect the function, polymerase complex formation or virion incorporation, and that fluorescent signals of rSeVLeGFP-infected cells represent the viral polymerase complex in association with vRNPs.

### LeGFP movement in live cells

**Figure 2:**
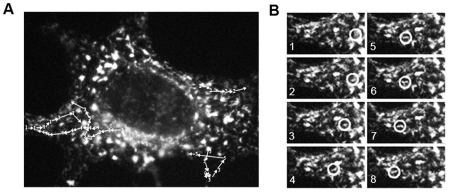
Live cell imaging of LeGFP particle movements. HeLa cells were infected with rSeVLeGFP for 18 h. Images were collected at 1 frame per sec, with an exposure time of 0.5 sec. (A) Typical trafficking of vRNPs. Positions of 5 different LeGFP signals every 4 seconds are shown. (B) Montage of the movement of LeGFP signal #4 is shown. The circles indicate the same LeGFP signal (#4) in (A) in successive frames. The images are selected frames from a video (Video S1).

To ascertain the type of movement vRNPs exhibit in live infected cells, we recorded the trafficking of LeGFP using digital video microscopy. Movement of vRNPs through the cytoplasm of infected cells was observed under fluorescent microscope, and images were collected at 1 frame per sec, with an exposure time of 0.5 sec (Figure 2A, B and Video S1). Capturing 1 frame per sec facilitated following the movement of distinct fluorescent particles traveling within the same focal plane. We observed a large number of small fluorescent dots traveling throughout the cytoplasm. These dots exhibited directional and saltatory movement. The small fluorescent dots also moved into and out of larger accumulations of fluorescent signal. Some small fluorescent dots were released from the large areas of accumulated vRNP and traveled in various directions. Most of the fluorescent dots traveling a long distance were small in size, and little movement of large aggregates was observed.

**Figure 3:**
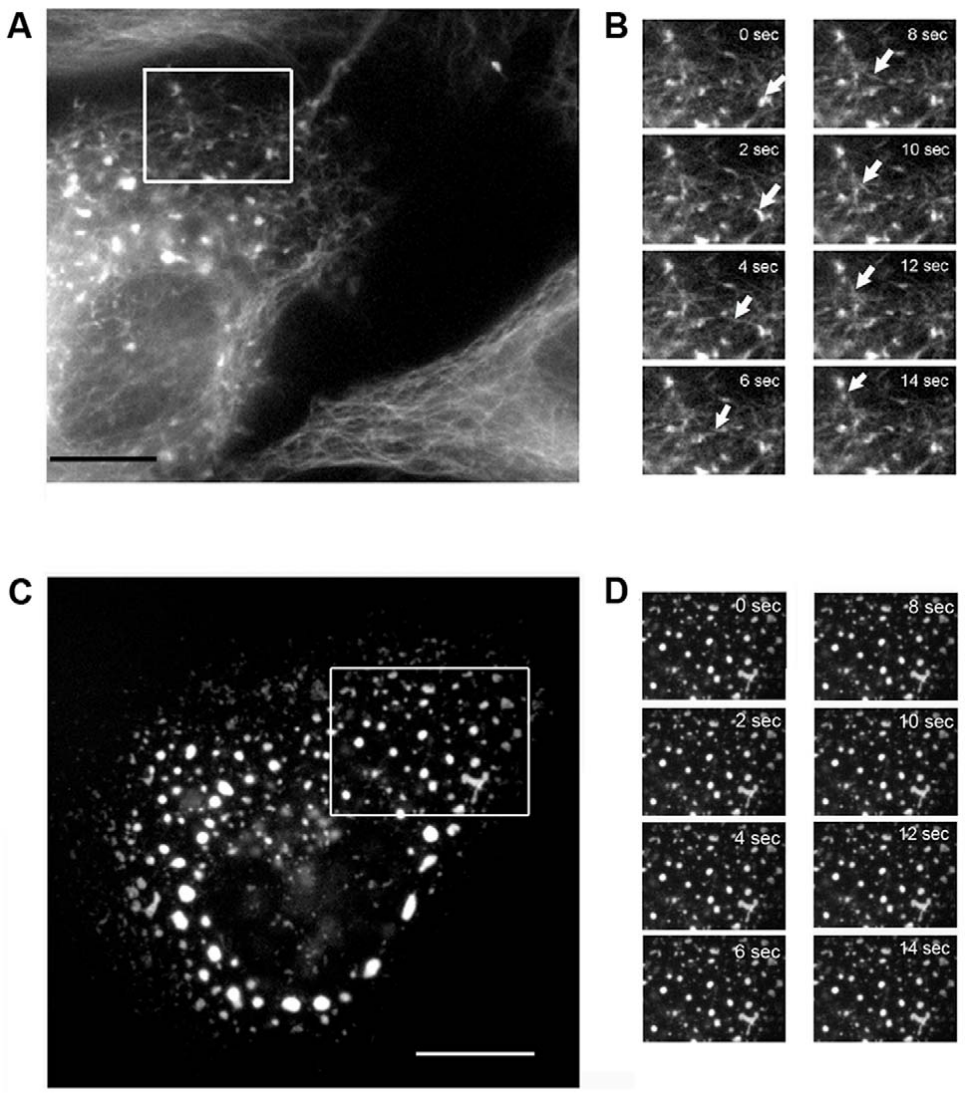
Tracking LeGFP movement in live cells along MT structures. HeLa cells were infected at MOI 0.5 and treated with 250 nM Tubulin Tracker Green at 18 h p.i. Images were collected by digital video microscopy. (A) Image of rSeVLeGFP-infected cell (left), which shows both LeGFP and microtubules. The white rectangle represents the area of the infected cell that was used to make the time-lapse series shown in (B). Bar, 10 µm. (B) Single frame images of LeGFP particle movement along MT structures over time. The arrows indicate movement of the same LeGFP particle along microtubules. (C) Effect of nocodazole treatment on LeGFP trafficking. Infected HeLa cells were treated with 10 µg/ml nocodazole for 1 h at 18 h p.i., and then stained with Tubulin Tracker Green. (D) Single frame images of nocodazole-treated cells, showing no movement of LeGFP.

The directional, saltatory movement of the fluorescent LeGFP particles is suggestive of microtubule (MT)-dependent transport, which has been reported with VSV [11]. To determine if LeGFPs move alongside MT structure, we infected HeLa cells with rSeVLeGFP, and at 18 h post infection, cellular MT structure was visualized with Tubulin Tracker Green, a microtubule binding protein (Taxol) labeled with Alexa Fluor 488, which allows live cell imaging of MTs (Figure 3 and Video S2). The cell at the bottom right of Figure 3A is uninfected and shows MT staining only, and the cell on the left of the panel exhibits a multitude of fluorescent LeGFP particles associated with MTs. Digital video microscopy analysis clearly showed vRNP movement along MTs. Figure 3B represents a successive frame analysis of vRNP movement, showing the trafficking of an LeGFP particle moving alongside MT structure. We tracked the movement of multiple particles to determine the average particle velocities. The average velocities of LeGFPs which traveled greater than 2 µm in length were 0.41–1.04 µm/sec, which is consistent with reported MT cargo movement of 0.2–2.7 µm/sec [26].

### Role of microtubules in trafficking vRNPs through the cytoplasm

To determine the role of MTs in vRNP trafficking, we treated infected cells with the MT destabilizing drug, nocodazole. Nocodazole was added to rSeVLeGFP-infected cells at 18 h post infection, and after 1 h treatment, movement of vRNP was recorded by digital video microscopy (Figure 3C, 3D and Video S3). In the nocodazole treated cells, LeGFP particles exhibited no movement, besides simple Brownian motion. Upon removal of the drug and replacement with fresh medium, the particles resumed their previously described movement (data not shown).

**Figure 4:**
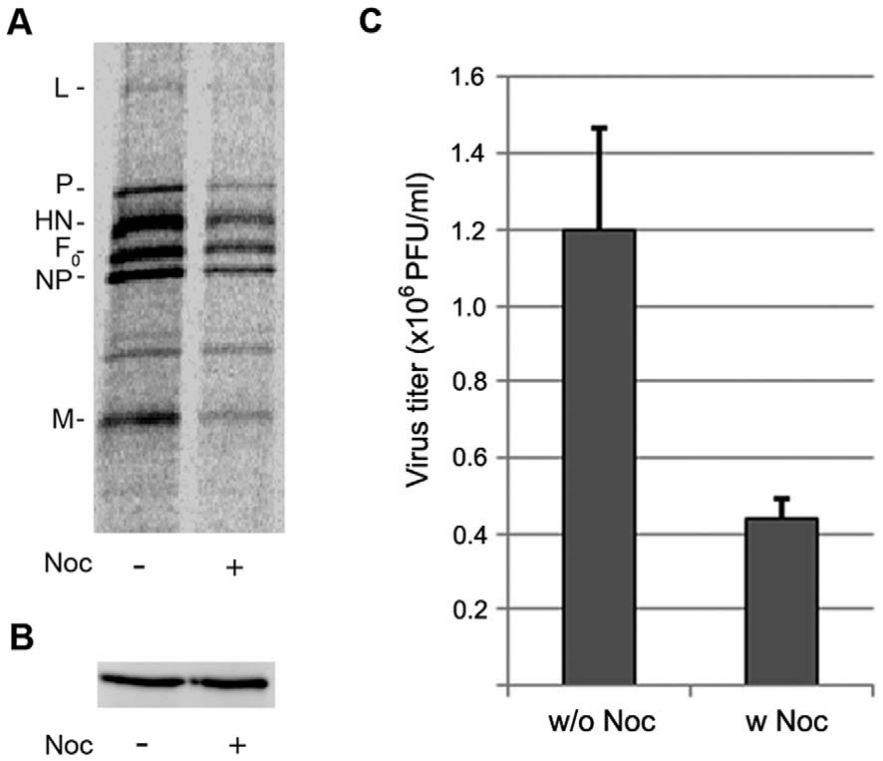
Nocodazole treatment does not affect protein synthesis, but reduces progeny virion production. (A) Progeny ^35^S-labeled SeV virions produced from infected cells cultured in the presence or absence of nocodazole were purified and analyzed by SDS-PAGE. (B) Level of NP proteins in nocodazole-treated or non-treated HeLa cells was compared by Western blotting using anti-NP Mab. Same amounts of cell lysates were used for comparison. (C) Supernatants from SeV-infected LLC-MK_2_ cells cultured in the presence or absence of nocodazole were collected and infectious viral titer was determined by plaque assay. Noc: nocodazole.

To further characterize the role of MT in SeV replication and assembly, infected cells were labeled with ^35^S-Met/Cys for 16 h in the presence or absence of nocodazole. The progeny virions released into the culture medium were collected, purified and analyzed by SDS-PAGE. As shown in Figure 4A, nocodazole treatment reduced production and release of progeny virions into the medium. Viral protein synthesis was not affected by nocodazole treatment (Figure 4B), suggesting the role of MT in viral assembly. Effect of nocodazole on virus production was also determined in LLC-MK_2_ cells. Cells infected with SeV for 4 h were either treated with or without nocodazole for 20 h, and the amounts of infectious viruses in medium were determined. Consistent with the result of virion production in HeLa cells, the amount of infectious virus was decreased 63% in the presence of nocodazole. These data indicate that vRNPs utilize MT structure for their translocation inside the cytoplasm, which is likely to be required for efficient assembly and release of progeny virions.

### Involvement of vesicular trafficking pathways in vRNP translocation

**Figure 5:**
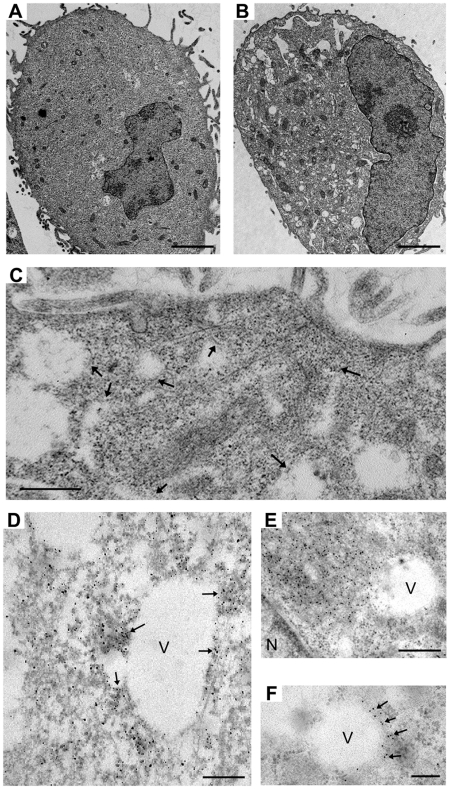
EM analysis of SeV-infected cells showing close association of vRNP to intracellular vesicles. HeLa cells were either uninfected (A) or infected (B) with SeV for 18 h at MOI 3.0. Ultra-thin sections were stained with uranyl acetate and lead citrate. Note that infected cell (B) contains many vesicles, which are not observed in uninfected cells (A). Bars, 2 µm. (C) EM image showing nucleocapsid-like structures close to or associated with vesicles. Arrows indicate nucleocapsid-like structures, many of which are associated with vesicles. Bar, 500 nm. (D) NP staining of SeV-infected HeLa cells. Fixed and permeabilized cells were reacted with anti-SeV NP cocktail and goat anti-mouse IgG HRP, followed by DAB staining. Arrows denote examples of NP localization visualized by DAB precipitate. Bar, 200 nm. (E and F) Detection of NP by EM using immuno-gold beads. Sections of infected cells were reacted with anti-SeV NP Mab, then with goat anti-mouse 12 nm gold beads. (E) A cellular vesicle located close to an accumulated NP. Bar, 500 nm. (F) NP localized around a vesicular structure. Bar, 200 nm. V, vesicles. N, nucleus.

To gain a detailed insight into nucleocapsid transport, we examined vRNP localization in infected cells by ultrastructural analysis using electron microscopy (EM). HeLa cells were infected with SeV or left uninfected for 18 h, and processed for EM analysis, as described in the Materials and Methods. Ultrathin sections of the epoxy resin embedded infected or uninfected cells were compared by transmission electron microscopy. Compared with the uninfected cells, SeV-infected cells exhibited a significant increase in the number of vesicles present in the cytoplasm (Figure 5A and B). Images of infected cells indicated many nucleocapsid-like structures located close to intracellular vesicles (Figure 5C). To confirm vRNP association to cellular vesicles, we performed immuno-EM analysis using anti-NP specific Mab. We did both pre-embedded labeling using horseradish peroxidase (HRP) and 3, 3′-Diaminobenzidine (DAB) staining and post-embedded labeling using immuno-gold beads. In SeV-infected cells, many NP signals were detected in areas around vesicles (Figure 5D, E and F). We observed vesicles close to the large vRNP accumulates (Figure 5E), as well as the vesicles associated with vRNPs around the vesicular membrane (Figure 5F). The uninfected cells, in contrast, did not have noticeable DAB staining or immuno-gold particles (data not shown). The heavy concentration of NP around vesicles in infected cells is likely to signify that vRNPs are utilizing these vesicles for transport to assembly sites for incorporation into virions.

**Figure 6:**
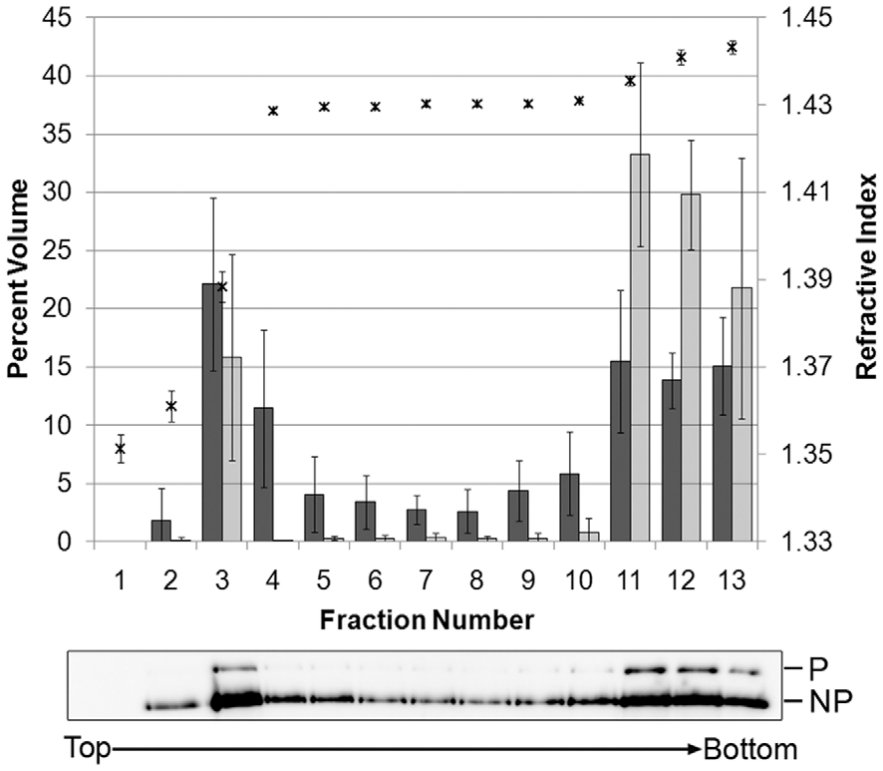
Membrane association of NP in infected cells. HeLa cells were infected with rSeVLeGFP for 12 h. The post nuclear supernatants were prepared in 85% sucrose resulting in a final concentration of 71.5% sucrose, which was layered with 55%, then 10% sucrose solutions. After centrifugation, thirteen 800 µl fractions were collected. Quantity of NP (dark gray bars) and P (light gray bars) in each fraction was determined by Western blot analysis using anti-SeV polyclonal antibody and analyzed using Quantity One software. The refractive index of each fraction is denoted by asterisks. Data are representative of 3 separate experiments. Image of one representative Western blot of NP and P is also shown below the graph.

Association of vRNP with cellular membrane vesicles was also determined by membrane flotation assay. Infected cell lysate in 71.5% sucrose solution was overlayed with 55% and 10% sucrose solutions, and after ultracentrifugation, 13 fractions were collected and the presence of NP in the fractions was determined by Western blot analysis using anti-SeV polyclonal serum. Approximately 35% of NP was recovered from membrane associated fractions (Figure 6, fractions 3 and 4), which seems to be a reasonable proportion based on the population of vRNPs in motion in live cells (Figure 2). These membrane-bound NP also fractionated with P, as detected by the polyclonal serum. Since many intracellular vesicles like endosomes, lysosomes, and secretory vesicles move along MTs [17], our overall results suggest that the vesicular trafficking pathway is involved in the translocation of vRNPs.

### Co-localization and concomitant movement of vRNP with Rab11a

**Figure 7:**
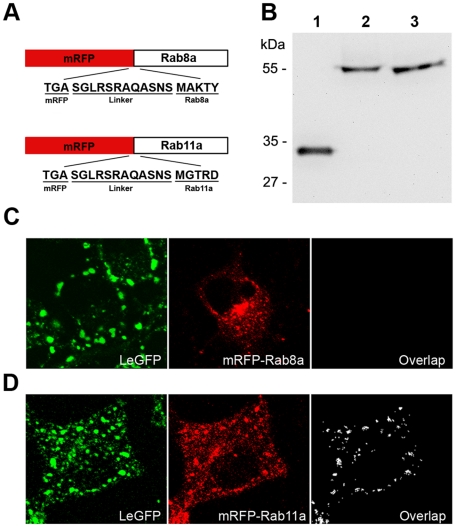
Co-localization of Rab11a with vRNP. (A) Diagram of constructs showing mRFPs fused to the N-terminus of Rab8a or Rab11a, including the linker sequence. (B) Western blot analysis of Rab constructs. HeLa cells were transfected with cDNAs expressing mRFP (lane 1), mRFP-Rab8a (lane 2) or mRFP-Rab11a (lane 3), and lysates were analyzed by Western blot using an anti-RFP antibody. (C and D) Co-localization of mRFP-Rab11a, but not mRFP-Rab8a with LeGFP. HeLa cells were infected with rSeVLeGFP and transfected with cDNAs that express (C) mRFP-Rab8a or (D) mRFP-Rab11a. Cells were fixed at 16 h after infection/transfection and observed using confocal microscopy. Left and middle panels show LeGFP and mRFP-Rab8a or -Rab11a, respectively, and right panels show overlapping degree of intensity, as determined by Olympus FV1000 software.

**Figure 8:**
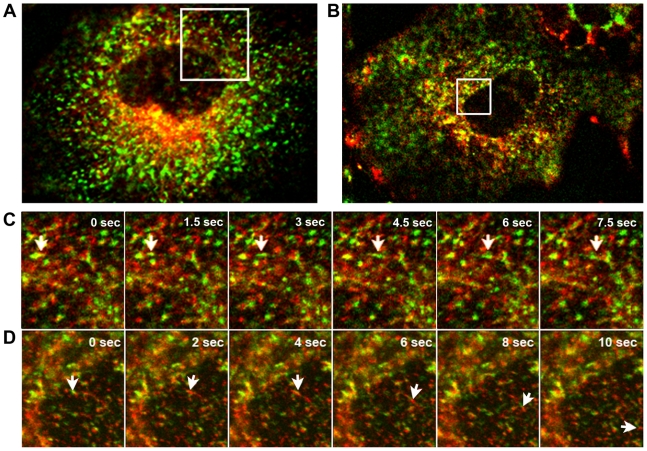
Concomitant movement of vRNP with mRFP-Rab11a or transferrin. (A and C) SeV LeGFP (green) in cells constitutively expressing mRFP-Rab11a (red). Montage illustrating movement of mRFP-Rab11a with LeGFP in boxed area of (A) is shown in (C). (B and D) HeLa cells infected with rSeVLeGFP were incubated with transferrin-Alexa 594. Montage images of concomitant movement of LeGFP (green) and transferrin (red) in boxed area of (B) are shown in (D).

Members of the Rab GTPase family serve as master regulators of vesicular membrane transport on both the exo- and endocytic pathways. Rab8 and Rab11 have been identified as regulators of vesicular trafficking by recycling endosomes or exocytosis from the trans-Golgi network to the cell surface [16]. To further characterize the vesicular trafficking pathway that vRNPs may utilize for translocation, we created two cDNA constructs, pmRFP-Rab8a and pmRFP-Rab11a, which express monomeric red fluorescent protein (mRFP) fused to Rab8a or Rab11a (Figure 7A). Expression of these fusion proteins was confirmed by Western blot analysis, showing the presence of appropriate size products and not mRFP alone in transfected cell lysates (Figure 7B). We determined the localization of the mRFP-Rab proteins in rSeVLeGFP-infected cells by confocal microscope. There was no co-localization observed between mRFP-Rab8a and LeGFP (Figure 7C). In sharp contrast, mRFP-Rab11a clearly co-localized with LeGFP (Figure 7D). Statistical analysis of the overlapping degree of intensity generated by the Olympus Confocal FV1000 Software shows 42% of the overall mRFP-Rab11a signals and 30% of the LeGFP signals are co-localized. The mRFP-Rab11a was associated with both small and large vRNP signals in the cytoplasm. We next visualized movement of SeV vRNPs along with mRFP-Rab11a in live cells by digital video microscopy performed on a confocal microscope. To facilitate the live cell analysis, we first established a HeLa cell line that constitutively expresses mRFP-Rab11a. HeLa-mRFP-Rab11a cells were infected with rSeVLeGFP, and we observed the movement of both mRFP-Rab11a and SeV vRNPs in live cells. Many of the mRFP-Rab11a were found to move concomitantly with small vRNP particles (Figure 8A, 8C and Video S4), in a manner consistent with movement along MTs. Together with the EM and membrane flotation assays, these results suggest that the vesicular trafficking pathway regulated by Rab11a is involved in vRNP trafficking.

### Movement of vRNPs with transferrin in live cells

Rab11 is well characterized for its role in regulating membrane traffic from the endocytic recycling compartment to either the plasma membrane or the trans-Golgi network [21], [27]. To further determine the involvement of recycling endosomes in vRNP trafficking, we visualized vRNP movement along with fluorescently labeled transferrin that binds to transferrin receptors, quintessential markers of recycling endosomes. Movement of the transferrin receptor along the recycling endosome pathway is known to be regulated by the activation of Rab11, which is required for direct recycling from the sorting endosome to the cell surface [28]. HeLa cells infected with rSeVLeGFP were incubated with fluorescently labeled transferrin, and trafficking of LeGFP and transferrin in live cells was recorded using confocal digital video microscopy. The transferrin movement was very similar to that of mRFP-Rab11a and vRNPs, which was directional and saltatory, indicative of translocation along MTs. In addition to individual movement, we detected concomitant movement of recycling transferrin with vRNP (Figure 8B and D, Video S5). These data provide additional corroboration that SeV vRNPs utilize recycling endosomes for transport from the cytoplasm to assembly sites at the plasma membrane.

## Discussion

The paramyxovirus infection is initiated by virus attachment, followed by membrane fusion at the plasma membrane and subsequent release of vRNP into the cytoplasm of the cell. The vRNP serves as a template for transcription and replication of the genome. With rSeVLeGFP that expresses L protein fused with eGFP (LeGFP), we could visualize the synthesis and intracellular movement of vRNPs in live infected cells. The LeGFP signals were detected as early as 8 h p.i., and at 12 h p.i., the signals were easily visualized with most forming tiny dots. It is unlikely that vRNPs are formed at particular limited locations in the cytoplasm because NP-P-L complexes appear all over the cytoplasm of infected cells (Figure 1D). There are many NP or P not co-localized with LeGFP (Figure 1D). However, over 90% and almost 80% of LeGFP co-localized with P and NP, respectively, suggesting that the majority of LeGFP in infected cells are associated with nucleocapsid complex. Because the level of LeGFP co-localization with NP was slightly less than that with P, it is likely that a small portion of the LeGFP exists as a complex with only P. Previous studies have demonstrated that P can bind NP alone, behaving as a chaperone for NP, preventing NP self-assembly into nucleocapsid-like complexes [29]–[30]. Therefore, it is reasonable to detect NP or P not associated with LeGFP in infected cells (Figure 1D). Although the majority of LeGFP was detected in association with NP and P, it is not known whether all the nucleocapsids contain viral genomic RNA in the complex. However, the NP-P complex is known to act as a substrate for viral RNA encapsidation. P and L complexes have also been suggested as necessary precursors to association with nucleocapsid template for replication [31]. Therefore, extensive co-localization of LeGFP with NP strongly suggests that the majority of LeGFP signals we observed represent vRNPs.

At an early stage of infection, we detected many LeGFP dots throughout the cytoplasm. Late after infection, large accumulations of LeGFP signals became evident (Figure 1C). Appearance of the accumulated vRNPs may indicate the presence of particular sites in the cytoplasm which provide an environment for enhanced formation of progeny vRNPs late after infection. Other negative strand RNA viruses also exhibit nucleocapsid accumulations in the cytoplasm of infected cells, such as Negri bodies, which are considered to be genome replication sites formed during the early stages of rabies virus infection. A recent study indicates that the initial large Negri bodies, of which only a couple appear per cell, gradually decrease in size with an increase in the appearance of many small nucleocapsid structures over the course of infection [32]. In contrast to rabies virus, numerous SeV vRNPs appear throughout the cytoplasm during the early stages of infection and gradually accumulate over time. It is therefore possible that SeV vRNPs accumulate at specific locations during their translocation. We observed small fluorescing dots (vRNP) coming out of the large clusters and moving in a directional manner. We also observed some small dots traveling long distances and merging into larger vRNP clusters (Figure 2 and Video S1). These varied movements of vRNPs going into and coming out of large vRNP accumulations may suggest the involvement of multiple steps in vRNP trafficking.

The directional movement strongly suggests that vRNPs utilize a specific intracellular trafficking pathway for their translocation, instead of moving by diffusion, which is severely restricted for macromolecular transport in the cytoplasm [33]–[34]. By staining MT in live rSeVLeGFP-infected cells, we were able to capture vRNP movement alongside MT, clearly showing vRNP trafficking through a MT-based mechanism (Figure 3A and B, Video S2). Nocodazole treatment restricted the movement of vRNP (Figure 3C and D, Video S3), and reduced production of progeny virions (Figure 4), supporting the idea that trafficking of SeV vRNPs through MT is involved in the assembly process of progeny virion formation at the plasma membrane. MT motors have been shown to catalyze the intracellular transport of many viral structures [35]–[36]. There are two alternative strategies viruses utilize for transport, either direct interaction of viral components with MT motors or hijacking cytoplasmic vesicular traffic [36]. It is possible that SeV vRNPs directly interact with kinesin motors and are transported by these motors on MT tracks; however, we were unable to detect direct interaction between vRNP and kinesin by co-immunoprecipitation (data not shown). Instead, our study using EM (Figure 5), as well as membrane flotation assay (Figure 6) strongly suggested that vRNPs utilize the vesicular trafficking system for transport to assembly sites. MT motors are known to drive the transport of intracellular vesicles within the cell. Rab proteins, which function as key regulators of intracellular trafficking have been linked to specific MT- or actin-based motor proteins [37]. We found that mRFP-Rab11a co-localize (Figure 7) and concomitantly move with vRNP (Figure 8C) in live cells, which also supports the involvement of intracellular vesicular trafficking regulated by Rab11a in vRNP translocation. Rab GTPases have been implicated in the regulation of membrane traffic, including vesicle budding, tethering/docking to their target compartments and in the interaction of vesicles with cytoskeletal elements. Rab proteins are anchored to lipid bilayers via C-terminal prenylation sites at cysteine residues [38], and regulate distinct intracellular transport steps through their interaction with effector proteins. Various Rab11 family of interacting proteins (Rab11-FIPs), which contain a highly conserved C-terminal Rab binding domain have been identified [39]-[40]. One of the Rab11 effector proteins FIP2 has been shown to interact with myosin Vb, an actin-based motor protein [41]. A ternary complex of Rab11-FIP2-myosin Vb is likely to provide the link between endosomes and the cytoskeleton to regulate the delivery of vesicular cargo to the plasma membrane. Similarly, evidence of direct binding between Rab11-FIP5/Rip11 and the MT-based motor Kinesin II was shown [42]. Rab11-FIP5/Rip11 is a Rab11-interacting protein that is localized to apical recycling endosomes in polarized epithelial cells [43] and regulates protein sorting and transport in non-polarized cells [42], suggesting interactions between the motor proteins and Rab11/Rab11-FIPs are required for endocytic apical transport.

Among the Rab proteins, Rab8a and Rab11a have been identified as being involved in recycling endosomes and Golgi-plasma membrane trafficking [16], [20]–[21], [27], [44]–[45]. However, a recent study indicates that Rab11a and Rab8a define different recycling pathways. Rab8a localize to a tubular network containing EHD1 and EHD3, which does not contain Rab11a. Live cell imaging also demonstrated distinct pathways for Rab11a and Rab8a vesicle trafficking [46]. We detected no co-localization of Rab8a with vRNP, in sharp contrast with the results of Rab11a, suggesting that the vesicular pathway regulated by Rab11a, but not Rab8a, is involved in vRNP trafficking. In fact, we detected vRNP movement in parallel with fluorescently-labeled transferrin that binds to the transferrin receptor, a quintessential marker of recycling endosomes (Figure 8D and Video S4). The movement of the transferrin receptor along the recycling endosome pathway is regulated by the activation of Rab11, which is required not only for endocytosis of transferrin into the recycling compartment but also for direct recycling of the sorting endosomes to the cell surface [28]. In polarized cells, Rab11 proteins and Rab11-FIPs regulate apical recycling endosomes, mediating the proper sorting and targeting of proteins during polarization [39], [47]–[48]. Since SeV is released from the apical surface of respiratory epithelial cells, vRNP trafficking regulated by Rab11 may facilitate efficient virus assembly.

Although little is known about the cellular machinery involved in the transport of paramyxovirus core structural proteins, the vesicular trafficking pathway has been implicated in transporting some components of enveloped RNA viruses to sites of viral assembly. Marburg virus VP40, the matrix protein, was shown to be associated with late endosomal compartments. VP40-positive membranes contained Lamp-1, a marker protein of late endosomes, and the transferrin receptor. Also, purified Marburg virus contained trace amounts of Rab11 [49]–[50]. Late endosomes were also shown to be the site of Marburg viral envelope formation [51]. In the case of respiratory syncytial virus (RSV), expression of a dominant negative form of myosin Vb or the C-terminal fragment of Rab11-FIP1 reduced viral yield released from the apical surface of polarized cells [52]. A dominant negative form of Rab11-FIP2 also reduced the supernatant-associated RSV titer, suggesting that Rab-FIPs are required for the budding of RSV [53]. It is not known whether Rab11 and Rab11-FIPs are involved in vRNP transport and assembly of RSV.

How SeV vRNPs associate with vesicles is an important question in understanding the process of viral assembly. It is possible that vRNPs are transported through interactions with other viral proteins, such as M or envelope glycoproteins. In SeV, the M protein has been shown to bind the viral glycoproteins, which are transported to the plasma membrane by means of the secretory pathway [3]. M also interacts with specific NP, and this specific interaction is required for incorporation of vRNP into progeny virions [4]. Because M plays a central role in viral assembly and budding, it is possible that M interacts with vRNP not only at the plasma membrane during virion formation, but also before vRNPs reach the assembly site. Further analysis of the interaction between viral proteins and Rab and Rab-associated proteins are required for understanding the mechanism of viral assembly.

## Materials and Methods

### Cells and viruses

LLC-MK_2_ (ATCC, CCL-7), HeLa (ATCC, CCL-2), HeLa T4^+^ [54] and 293T [55] cells were cultured in Dulbecco's modified Eagle's medium (DMEM) with 10% fetal calf serum (FCS). SeV strain Enders, rSeVLeGFP, and rSeV-eGFP were grown in LLC-MK_2_ cells in DMEM supplemented with acetylated trypsin (2 µg/ml). vTF7.3 [56] was grown in HeLa T4^+^ cells.

### cDNA synthesis and cloning

The full genome cDNA of rSeVLeGFP (pSeVLeGFP) was constructed as follows. The cDNA containing the L protein C-terminus was subcloned from pSeV(E) [57] to plasmid pTF1 [58] and an *FseI* site was created at the end of the L coding region using the QuikChange Mutagenesis Kit (Stratagene). The eGFP gene was amplified by PCR from plasmid pEGFP-N1 (Clontech) using primers containing *FseI* sites flanking the gene, and was inserted into the L gene fragment in pTF1 at the *FseI* site. The L gene fused to the eGFP gene was then subcloned back to pSeV(E). The cDNA of rSeV-eGFP (pSeV-eGFP), which expresses non-fused eGFP was constructed by inserting the eGFP gene amplified from pEGFP-N1. The primers used for PCR included transcription termination and initiation sites, as well as the restriction (*NotI*) site used for the insertion of the gene into pSeV(+)N [59]. Rab8a (NM_005370) and Rab11a (NM_004663) genes were cloned from total RNA extracted from HeLa cells. RT-PCR was performed using Rab8a and Rab11a specific primers, which also include *EcoRI* and *KpnI* sites for subcloning the genes to the pmRFP-C1 vector (Clontech).

### Rescue of rSeVLeGFP and rSeV-eGFP

The recombinant SeVs were rescued as described previously [60]. Briefly, 293T cells infected with vTF7.3 were transfected with 2 µg of pSeVLeGFP or pSeV-eGFP together with supporting plasmids pTF1SeVNP (1 µg), pTF1SeVP (1 µg), and pTF1SeVL (0.1 µg) by Lipofectamine 2000 (Invitrogen). After 36 h incubation in DMEM supplemented with 0.15% bovine serum albumin plus araC (40 µg/µl), the cells were treated with trypsin and overlayed onto LLC-MK_2_ cells and cultured in DMEM containing trypsin. The rescued virus was plaque purified in LLC-MK_2_ cells, and the stock virus was grown in LLC-MK_2_ cells.

### Establishment of HeLa cells constitutively expressing Rab11a- or Rab8a-mRFP

The mRFP, mRFP-Rab8a, and mRFP-Rab11a genes were subcloned from those in pmRFP-C1 plasmids into the pCAGGS-HygR vector, which contains a hygromycin resistance gene under IRES. HeLa cells (obtained from ATCC) were transfected with the cDNAs using CaPO_4_, and hygromycin resistant colonies were selected and grown in DMEM containing 8% FCS.

### Western blot analysis

To detect expression of LeGFP or eGFP, infected cell lysates were prepared using TNE buffer (10 mM Tris [pH 7.4], 150 mM NaCl, 0.5% NP-40, 1 mM EDTA). Proteins in the SDS-PAGE were electrotransferred onto PVDF membranes and probed with specific Mab against GFP (Covance). To detect the expression of Rab8a or Rab11a fused to mRFP, HeLa cells were transfected with the cDNAs and cell lysates were prepared after 24 h using TNE buffer and analyzed using anti-RFP antibody (Rockland), as described above.

### Immunofluorescence assays

For the co-localization assay of SeV NP, P or F with LeGFP, HeLa cells were infected with rSeVLeGFP at MOI 0.8. After 24 h, cells were fixed with 4% paraformaldehyde (PFA) for 15 min and permeabilized with 0.1% Triton X-100 for 10 min at room temperature (RT). SeV NP, P and F proteins were detected by reaction with anti-SeV NP, anti-SeV P or anti-SeV F Mab followed by goat anti-mouse Texas Red (Molecular Probes).

### Co-localization of Rab8a and Rab11a with rSeVLeGFP

HeLa cells were infected with rSeVLeGFP for 1 h, then transfected with either pmRFP-Rab8a or pmRFP-Rab11a, using Lipofectamine 2000 (Invitrogen). After overnight incubation at 34°C, cells were fixed with 4% paraformaldehyde and analyzed by confocal microscopy using a 63X oil immersion objective on an Olympus FV1000 confocal microscope. The lasers utilized were 488 (GFP) at 3% and 559 (RFP) at 2.3% with Kalman Line = 4. The data were analyzed using the Olympus FV1000 software (v. 1.7c).

### Live cell tracking analysis

HeLa cells in a ΔT35 dish (Bioptechs) were infected with rSeVLeGFP for 18 h, then the movement of LeGFP was recorded using a Leica DMIRB inverted fluorescence microscope equipped with Image-Pro Plus software (Mediacybernetics) while maintaining the cells at 37°C on a ΔTC3 temperature-controlled stage. The video image data was analyzed using NIH ImageJ software. For visualizing movement of LeGFP along microtubule structures, infected cells were treated with 250 nM Tubulin Tracker Green (Molecular Probes) for 30 min at 37°C in PBS(+), and imaged as above. To observe mRFP-Rab11a movement with vRNPs, the HeLa-mRFP-Rab11a cells were infected with rSeVLeGFP for 16 h, and observed using an Olympus FV1000 confocal microscope. A 63X oil immersion objective was utilized, with a 488 (GFP) laser at 9% and a 559 laser (RFP) at 10%. The pinhole was open to 240, and the resolution was set at 640×640, 2 µs/pixel at 1.5 frames/sec. For the visualization of concomitant movement of transferrin along with vRNPs, HeLa cells infected with rSeVLeGFP for 18 h were treated with 5 µg/ml transferrin conjugated to Alexa 594 (Molecular Probes, Invitrogen) for 30 minutes at 37°C. Cells were analyzed by confocal microscopy, using a 63X oil immersion objective on an Olympus FV1000 confocal microscope. The lasers utilized were 488 (GFP) at 3% and 559 (RFP) at 1.5%. Resolution used was 1600×1600, 12.5 µs/pixel, with a step size of 4.5 µm at 1.5 frames/sec. The data were analyzed using the Olympus FV1000 software (v. 1.7c). Additional video editing was performed using Adobe After Effects CS3 (v. 8.0.2).

### Effect of nocodazole on virus replication and production

HeLa cells infected with SeV for 24 h were cultured in labeling medium containing 100 µCi of ^35^S-Met/Cys (PerkinElmer) in the presence or absence of nocodazole (20 µg/ml) for 16 h. Progeny virions in culture supernatants were purified by ultracentrifuge over 40% glycerol in PBS, and analyzed by SDS-PAGE. Level of NP proteins in infected cells was determined by Western blotting using anti-SeV NP monoclonal antibody. For titration of infectious virus released into the culture medium, we infected LLC-MK_2_ cells with SeV at an MOI of 1, and after 4 h incubation, culture medium was replaced with medium with or without nocodazole at 5 µg/ml. After 20 h incubation, viruses in the medium were titrated by plaque assay in LLC-MK_2_ cells.

### Electron Microscopy

HeLa cells were infected with rSeVLeGFP or left uninfected for 18 h, and fixed in 4% PFA. The cells were then post-fixed with 1% OSO_4_ and ultra-thin sections were stained using uranyl acetate and lead citrate. The images were collected using a Hitachi 7100 transmission electron microscope. For immunoelectron microscope analysis using 3, 3′-Diaminobenzidine (DAB) staining, fixed and permeabilized cells were treated with blocking solution (0.8% BSA, 0.1% fish gelatin, 5% normal horse serum, and 0.01% Triton X-100 in PBS(+)) and reacted with anti-SeV NP Mab cocktail, followed by incubation with goat anti-mouse IgG horseradish peroxidase (HRP). After washing, the cells were stained using the DAB plus substrate kit (Zymed Laboratories, Inc.). The samples were silver enhanced, treated with 1% osmium and embedded in Epon. For the immuno-gold reaction, cells were fixed with 4% PFA and embedded in Lowicryl low melt resin. The cells were sectioned, placed on grids, and reacted with anti-SeV NP and goat anti-mouse 12 nm gold beads (Jackson Laboratories).

### Membrane Flotation Assay

Assay was performed as previously [61]. Briefly, cells infected with rSeVLeGFP for 12 h were lysed with hypotonic buffer and dounce homogenized. Supernatant after brief spin was mixed with 85% sucrose in TNE buffer, resulting in a 71.5% final sucrose concentration, which was overlayed by 55% and 10% sucrose in TNE buffer. The samples were centrifuged 18 h at 28,000 rpm (∼100,000×g) in an SW41 rotor at 4°C, and thirteen 800 µl fractions were collected. NP and P in each fraction were quantified by Quantity One image analysis software (Bio-Rad Laboratories) after Western blotting, using anti-SeV polyclonal serum and rabbit anti-guinea pig IgG HRP. The refractive index of the fractions was determined by hand-held refractometer (R-5000, Atago, U.S.A.).

## Supporting Information

Video S1 Movement of LeGFP in HeLa cells. Live cells infected with rSeVLeGFP were observed using a Leica DMIRB inverted fluorescence microscope equipped with Image-Pro Plus software. The movements of LeGFP were recorded while maintaining the cells at 37Â°C.
(5.80 MB MOV)

Video S2 Trafficking of vRNP along MT. rSeVLeGFP-infected cells were treated with 250 nM Tubulin Tracker Green and the movement of LeGFP was recorded as described in Video S1. Arrows indicate vRNP movements along MT.
(7.58 MB MOV)

Video S3 Nocodazole treatment restricts LeGFP trafficking. HeLa cells infected with rSeVLeGFP were treated with 10 ug/ml nocodazole for 1 h at 18 h post infection and the movement of LeGFP was recorded, as described for Video S1.
(9.95 MB MOV)

Video S4 Concomitant movement of vRNPs with mRFP-Rab11a. HeLa-mRFP-Rab11a cells were infected with rSeVLeGFP and live cell analysis was performed using an Olympus FV1000 confocal microscope. Circles indicate LeGFP and mRFP-Rab11a moving together in the cytoplasm.
(7.33 MB MOV)

Video S5 Movement of vRNPs with transferrin. HeLa cells were infected with rSeVLeGFP and transferrin-Alexa 594 was added. Trafficking of both transferrin-Alexa 594 and vRNPs was observed in live cells using the confocal microscope.
(4.52 MB MOV)
